# Electromagnetic Analysis and Experimental Study of Laminated Mn-Zn Toroidal Ferrite Cores for High-Frequency Inductance and Impedance Enhancement

**DOI:** 10.3390/mi17010043

**Published:** 2025-12-29

**Authors:** Penghui Guan, Yong Ren, Chunhua Tang, Li Wang, Bin Luo, Yingcheng Lin

**Affiliations:** 1 State Key Laboratory of Intelligent Vehicle Safety Technology, Chongqing 400020, China; guanph@changan.com.cn (P.G.); tangch@changan.com.cn (C.T.); wangli15@changan.com.cn (L.W.); 2 School of Microelectronics and Communication Engineering, Chongqing University, Chongqing 401331, China; renyong@cqu.edu.cn

**Keywords:** electromagnetic field, high-frequency inductor, laminated structure, Mn-Zn ferrite

## Abstract

To achieve high-frequency inductance and impedance enhancement for effective electromagnetic interference (EMI) mitigation in power electronics, this paper presents an electromagnetic analysis and experimental study of laminated Mn-Zn toroidal ferrite cores. The electromagnetic field is analyzed using a 2D analytical solution based on a simplified Cartesian approximation. Although neglecting curvature, this approach enables efficient eigenfunction expansion and is rigorously validated against cylindrical finite difference (FDM) and 3D finite element (FEM) benchmarks. The results demonstrate that lamination effectively interrupts eddy current loops; notably, a four-layer structure increases the resonant frequency by approximately 2.8 times compared to a monolithic core. Experimental measurements confirm that this design significantly mitigates the skin effect and extends the stable frequency bandwidth. This study establishes a validated, computationally efficient methodology for optimizing core geometries to prevent impedance degradation.

## 1. Introduction

In the field of high-frequency electromagnetic interference (EMI) conductive suppression [1,2,3], Mn-Zn ferrite materials have been widely adopted for use in filters, inductors, common-mode chokes, and shielding applications due to their excellent magnetic permeability and cost-effectiveness. However, the increasing demand for a high efficiency and power density in power conversion systems has caused the operating frequencies of advanced wide-bandgap semiconductor devices to shift into the MHz range [4]. This transition has posed significant challenges to traditional ferrite core filter designs, especially in applications involving toroidal magnetic structures, where the performance degradation becomes more pronounced. Studies have shown that at high frequencies, the impedance performance of ferrite cores deteriorates markedly. This is primarily due to the reduced frequency-dependent decline in permeability, as well as the magnitude and phase of impedance [5]. Consequently, traditional ferrite cores are limited in their ability to suppress high-frequency EMI noise peaks, restricting their applicability in modern electromagnetic compatibility (EMC) solutions.

**Figure 1:**
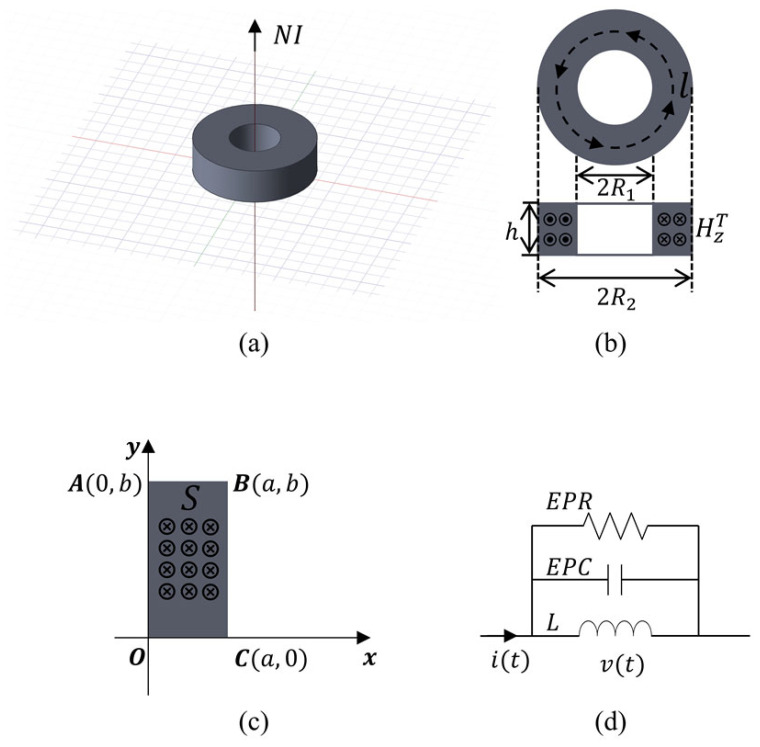
Geometry and equivalent circuit of the toroid ferrite core. (**a**) Ferrite core with axial excitation current passing through the center hole; (**b**) top and side views showing the toroid geometry and the induced magnetic field; (**c**) 2D cross-sectional Cartesian coordinate used for field calculations; (**d**) equivalent RLC parallel circuit.

As illustrated in Figure 1d, the impedance characteristics of ferrite cores with rectangular cross-sections can be modeled equivalently by an RLC parallel circuit [1,6,7]. At a high frequency, the inductive impedance is significantly affected by the equivalent parasitic capacitance (EPC) associated with the LC resonant frequency [8]. Even under the assumption of a significant magnetic field distribution, the EPC still plays a non-negligible role in shaping the overall impedance response [9]. The EPC primarily reflects the energy of the electric field within the ferrite core and is closely tied to the relative permittivity of ferrite materials. When the relative permittivity becomes significant, especially in Mn-Zn ferrite, the corresponding EPC dominates the core’s high-frequency behavior.

A method to ensure a uniform distribution of the incident magnetic field throughout a circular-cross-section core was proposed in [6], which involves external winding around the core and allows the equivalent parasitic capacitance (EPC) and the first resonant frequency to be derived analytically. This allows the EPC (also referred to as magnetized capacitance) and the first resonant frequency to be derived analytically. However, this assumption is invalid for the rectangular cross-section, as the induced eddy current field and the incident magnetic field are not uniformly aligned, particularly when the magnetic field strength varies significantly with the radial or axial position. An analytical method for one-dimensional magnetic field distribution in thin ferrite cores was proposed in [10] under the assumption of a weak diffusion effect, providing insights into eddy current-induced losses. However, this method assumes that the eddy field is purely radial. Based on the simulations in this work, a non-negligible axial component was observed, suggesting that such an assumption lacks sufficient accuracy.

Additionally, as the operating frequency increases, the initial reduction in permeability of Mn-Zn ferrite is caused by a domain wall relaxation phenomenon. This mechanism is directly influenced by the material’s microstructure, as grain boundaries impede the movement of domain walls, preventing them from keeping pace with the rapidly oscillating magnetic field [11]. At a high frequency, skin effects, due to the eddy current and dimensional resonance, emerge inside the core, producing flux crowding and highly non-uniform magnetic field distributions whose wavelengths are much smaller than the core’s cross-sectional dimensions [12]. This non-uniformity reduces both impedance and filtering effectiveness [12,13]. To mitigate these effects, [14] introduced a ferrite laminated configuration. Experimental evidence confirms that this structure markedly enhances high-frequency impedance, with the improvement becoming more pronounced as the number of laminations increases. Theoretical and energy-based analyses of the high-frequency field distribution and eddy current loss have since been refined in [6] and [12,13,14]. The efficacy of the lamination technique extends beyond ferrites; indeed, recent work [15] has demonstrated that next-generation laminated nanocrystalline cores can also achieve significant enhancements in both magnetic properties and thermal stability for high-frequency power electronics.

Although numerical solvers such as the finite element method (FEM) deliver high accuracy, they are less efficient and transparent in revealing physical mechanisms than the analytical approach adopted in this study. Furthermore, dimensional resonance and skin effects mean that the single-parameter data in commercial datasheets is insufficient for accurately modeling and simulating with complex permeability and permittivity [7,16,17]. To address this shortcoming, a technique based on impedance extraction was proposed in [18], enabling the simultaneous acquisition of both intrinsic parameters. Although this method has been successfully employed in subsequent studies [19,20], it necessitates customized test fixtures. Furthermore, the extracted material parameters have yet to be directly integrated into numerical simulations.

To address the limitations of ideal theoretical models and the computational burden of 3D simulations, this paper proposes a practical design methodology for laminated toroidal cores. Distinct from prior theoretical or purely numerical studies [7] and [12,13,14], this work fills the gap between analytical derivation and engineering application through the following contributions:

1. Integrate Modeling with Experimental Parameters: We establish a method to integrate experimentally extracted, frequency-dependent complex permeability into a 2D analytical solution. This approach bridges the gap between theoretical prediction and actual material performance, ensuring a high accuracy for commercial Mn-Zn ferrites.
2. Efficient 2D Analytical Solution: A closed-form solution based on eigenfunction expansion is derived, overcoming the accuracy deficits of 1D approximations while maintaining computational speeds orders of magnitude faster than FEM.
3. Quantified Design Guideline: This study experimentally validates the efficacy of lamination, quantifying a 2.8-times increase in resonant frequency for a four-layer structure, which provides a concrete, validated strategy for high-frequency magnetic component design.

The rest of this article is structured as follows. Section 2 provides a systematic derivation of the analytical solution for the magnetic and electric fields within ferrite cores, taking into account eddy current effects and energy computation. Section 3 compares the accuracy and efficiency of three solution methods for solving this class of partial differential equations (PDEs). The advantages of the analytical solution in terms of computational speed are demonstrated, along with validation through numerical simulations. Section 4 investigates how high-frequency inductance can be enhanced in laminated ferrite core structures, using both simulation and experimental validation. The intrinsic complex permeability and permittivity of the materials are extracted experimentally and incorporated into the analytical model for inductance prediction. The calculated results agree well with the measurements, thus confirming the accuracy and engineering feasibility of the proposed method for predicting the high-frequency performance of laminated inductors. Finally, the conclusions are drawn in Section 5.

## 2. Analytical Solutions of Magnetic and Electric Fields

**Figure 2:**
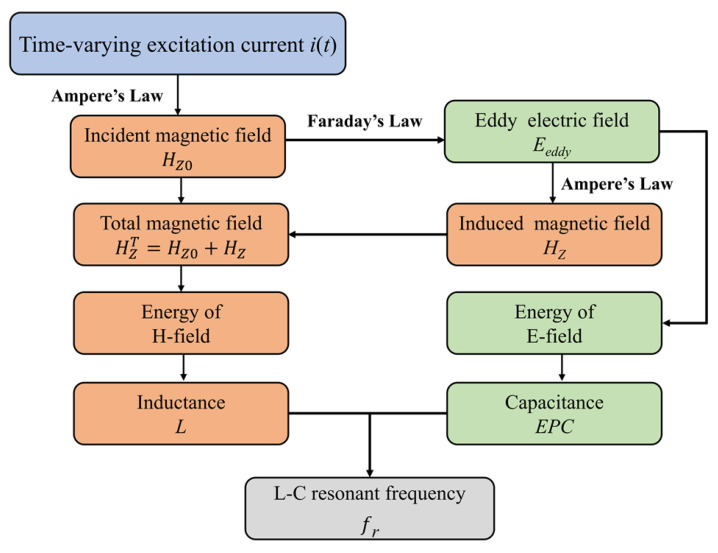
Coupled mechanism between magnetic and eddy electric fields inside the ferrite core.

As illustrated in Figure 2, the internal magnetic and eddy electric fields are coupled and influenced inside the ferrite core. The time-varying magnetic field generated by the alternating current induces an eddy electric field. This gives rise to an induced magnetic field, resulting in magnetic field superposition. The eddy current field also contributes to energy loss, which is commonly represented by the effective parallel capacitance. This parameter reflects the system’s ability to store or dissipate energy, thereby affecting its resonant behavior. Therefore, it is essential to consider this magnetic–electric interaction when analyzing the resonance frequency and electromagnetic energy transfer process in an LC resonant network.

As shown in Figure 1c, for the rectangular cross-section of the core, the total magnetic and electric fields inside the core satisfy Maxwell’s equations:

(1) $$\begin{array}{c} \nabla \times H=J=\left(\sigma +j\omega \varepsilon \right)E,\; \end{array}$$

(2) $$\begin{array}{c} \nabla \times E=-j\omega \mu H. \end{array}$$

The complex permeability and permittivity are expressed as $\mu =u_{0}(\mu_{r}^{\prime }-j\mu_{r}^{\prime \prime })$ and $\varepsilon =\varepsilon_{0}(\varepsilon_{r}^{\prime }-{j\varepsilon }_{r}^{\prime \prime })$, respectively. Taking the curl of both sides of Equation (1), applying the vector identity $\nabla^{2}H=\nabla \left(\nabla \cdot H\right)-\nabla \times (\nabla \times H)$, and using ∇·H=0, the vector equation for the magnetic field is

(3) $$\begin{array}{c} \nabla^{2}H+k^{2}H=0,\;k^{2}=-j\omega \mu \left(\sigma +j\omega \varepsilon \right). \end{array}$$

Considering the high permeability of the Mn-Zn ferrite core and the toroidal symmetry of the excitation, the magnetic flux is predominantly confined within the core cross-section along the azimuthal direction. Consequently, the magnetic field intensity is assumed to have only a z-component ($H=H_{z}\hat{z}$), while the radial and axial components are negligible. This simplification allows the vector wave equation to be reduced to a scalar form without loss of physical accuracy. The total magnetic field within the magnetic core can be decomposed into an incident field, which is generated by the excitation current, and an induced field, which is caused by eddy currents in the ferrite. Consequently, the total field can be expressed as $H_{z}^{T}=H_{z0}(x)+H_{z}(x,y)$, where $H_{z0}$ (seen in Figure 2) represents the incident magnetic field generated by an ideal AC excitation current (i.e., not affected by the ferrite), and can be approximated using Ampere’s law as $H_{z0}\left(x\right)=\frac{NI}{2\pi (x+R_{1})}$. The term $H_{z}$ (also seen in Figure 2) represents the induced magnetic field inside the core, which remains to be solved. Substituting this decomposition into the inhomogeneous Helmholtz equation yields the governing equation for the induced field:

(4) $$\begin{array}{c} \nabla^{2}H_{z}+k^{2}H_{z}={k^{2}H}_{z0}. \end{array}$$

To derive a computationally efficient closed-form solution using the method of separation of variables, the toroidal core geometry is approximated as a rectangular cross-section in the Cartesian coordinate system. In this approximation, the radial curvature terms (i.e., 1/r and $1/r^{2}$) in the Laplacian operator are neglected. This simplification transforms the cylindrical Helmholtz equation into a standard Cartesian form, enabling the electromagnetic field to be expanded continuously in terms of distinct eigenfunctions.

By expanding the toroidal core’s cross-section into a rectangular domain, 0≤x≤a and 0≤y≤b in the Cartesian coordinate, where the magnetic core thickness is defined as *b* (also denoting the core height). Because the outer surfaces are subject only to the incident magnetic field (and the tangential field component is continuous), the induced magnetic field satisfies homogeneous Dirichlet boundary conditions:

(5) $$\begin{array}{c} {\;H}_{z}\left(0,y\right)=H_{z}\left(a,y\right)=H_{z}\left(x,0\right)=H_{z}\left(x,b\right)=0. \end{array}$$

Physically, this is justified by the fact that eddy currents form closed loops within the cross-section. According to Ampere’s Law, the net current of these closed loops integrated over the cross-section is zero; thus, they do not contribute to the magnetic field at the boundary, leaving the surface field determined solely by the external excitation. We define the total magnetic field as $u\left(x,y\right)=H_{z}\left(x,y\right)$ and the source term as $f\left(x,y\right)=\frac{k^{2}NI}{2\pi \left(x+R_{1}\right)}$. Based on the standard Laplace operator ($\nabla^{2}=\partial_{xx}+\partial_{yy}$), (4) and (5) can be rewritten as the following Poisson-type partial differential equation (PDE):

(6) $$\begin{array}{c} \left(\nabla^{2}+k^{2}\right)u\left(x,y\right)=-f\left(x,y\right),\;\;{u|}_{\partial \Omega }=0. \end{array}$$

The eigenfunction expansion method is considered to solve this equation. Assuming that $\left(\nabla^{2}+k^{2}\right)u=0$ admits separable solutions, the eigenfunction $\psi_{mn}(x,y)$ and the corresponding eigenvalue $k_{mn}^{2}$ satisfy

(7) $$\begin{array}{c} \nabla^{2}\psi_{mn}\left(x,y\right)+k_{mn}^{2}\psi_{mn}\left(x,y\right)=0. \end{array}$$

By applying the method of separation of variables under Dirichlet conditions, the normalized eigenfunction and eigenvalue are given by

(8) $$\begin{array}{c} \;\psi_{mn}\left(x,y\right)=\sqrt{\frac{4}{ab}}\sin\left(\frac{m\pi x}{a}\right)\sin\left(\frac{n\pi y}{b}\right), \end{array}$$

(9) $$\begin{array}{c} k_{mn}^{2}={\left(\frac{m\pi }{a}\right)}^{2}+{\left(\frac{n\pi }{b}\right)}^{2}\;\;m,n=1,2,\cdots . \end{array}$$

Since Equation (4) is a non-homogeneous Helmholtz equation, the Green function method is introduced in the next step to facilitate the analytical solution:

(10) $$\begin{array}{c} u\left(x,y\right)=\iint_{\Omega }G\left(x,y|w,v\right)f\left(x,y\right)dwdv, \end{array}$$

in which $G\left(x,y|w,v\right)$ is the Green function corresponding to the differential operator $\left(\nabla^{2}+k^{2}\right)$, which satisfies the following equation:

(11) $$\begin{array}{c} \;\;\;\;\;\;\;\;\;\;\;\;\;\;\;\;\left(\nabla^{2}+k^{2}\right)G\left(x,y|w,v\right)=\\ \;\;\;\;\;\;\;\;\;\;\;\;\;\;\;\;\delta \left(x-w\right)\delta \left(y-v\right),\;\;{G|}_{\partial \Omega }=0. \end{array}$$

The Green function can be expanded in terms of the eigenfunction series as

(12) $$\begin{array}{c} G\left(x,y|w,v\right)=\sum_{m,n}A_{mn}\left(w,v\right)\psi_{mn}\left(x,y\right). \end{array}$$

Substituting Equation (12) into the left-hand side of Equation (11), and according to the eigenfunction identity from Equation (7), we obtain

(13) $$\begin{array}{c} \left(\nabla^{2}+k^{2}\right)G=\sum_{m,n}A_{mn}\left(w,v\right)\left(\nabla^{2}+k^{2}\right)\psi_{mn}\left(x,y\right)=\\ \;\;\;\;\;\;\;\;\;\;\;\;\;\;\;\;\;\;\;\sum_{m,n}A_{mn}\left(w,v\right)\left(-k_{mn}^{2}+k^{2}\right)\psi_{mn}\left(x,y\right). \end{array}$$

Meanwhile, the right-hand side of Equation (11) can be expanded as

(14) $$\begin{array}{c} \delta \left(x-w\right)\delta \left(y-v\right)=\sum_{m,n}\psi_{mn}\left(x,y\right)\psi_{mn}\left(w,v\right). \end{array}$$

By comparing both sides of Equations (13) and (14), the coefficients can be identified as

(15) $$\begin{array}{c} A_{mn}\left(w,v\right)=\frac{\psi_{mn}\left(w,v\right)}{k_{mn}^{2}-k^{2}}.\; \end{array}$$

Substituting Equation (15) back into Equation (12) completes the closed-form Green function expression, enabling the computation of the total magnetic field distribution:

(16) $$\begin{array}{c} G\left(x,y|w,v\right)\;=\sum_{m=1}^{\infty }\sum_{n=1}^{\infty }\frac{\psi_{mn}\left(x,y\right)\psi_{mn}\left(w,v\right)}{k_{mn}^{2}-k^{2}}. \end{array}$$

Therefore, the Green function can be written explicitly as follows:

(17) $$\begin{array}{c} \;\;\;\;\;\;\;\;\;\;\;\;\;\;\;\;G\left(x,y|w,v\right)=\frac{4}{ab}\cdot \\ \;\;\;\;\;\;\;\;\;\;\;\;\;\;\;\;\;\;\;\;\;\sum_{m=1}^{\infty }\sum_{n=1}^{\infty }\frac{\sin\left(\frac{m\pi x}{a}\right)\sin\left(\frac{\mathrm{m\pi w}}{a}\right)\sin\left(\frac{n\pi y}{b}\right)\sin\left(\frac{n\pi v}{b}\right)}{{\left(\frac{m\pi }{a}\right)}^{2}+{\left(\frac{n\pi }{b}\right)}^{2}-k^{2}}. \end{array}$$

By substituting Equation (17) into Equation (10), the expression for the induced magnetic field $H_{z}$ becomes

(18) $$\begin{array}{c} \begin{array}{c} \;H_{z}\left(x,y\right)=\int_{0}^{a}\int_{0}^{b}\frac{4}{ab}f\left(w,v\right)\\ \;\;\;\;\;\;\;\;\;\;\;\;\;\;\;\;\;\;\;\;\;\sum_{m=1}^{\infty }\sum_{n=1}^{\infty }\frac{\sin\left(\frac{m\pi x}{a}\right)\sin\left(\frac{\mathrm{n\pi y}}{b}\right)\sin\left(\frac{m\pi w}{a}\right)\sin\left(\frac{n\pi v}{b}\right)}{{\left(\frac{m\pi }{a}\right)}^{2}+{\left(\frac{n\pi }{b}\right)}^{2}-k^{2}}dwdv,\; \end{array} \end{array}$$

where the excitation term is $f(w,v)=k^{2}NI/2\pi (w+R_{1})$.

Therefore, the total magnetic field $H_{z}^{T}$ is the superposition of the incident magnetic field $H_{z0}$ (generated by the current) and the induced magnetic field $H_{z}$ (generated by eddy currents), and it can be expressed as follows:

(19) $$\begin{array}{c} \begin{array}{c} H_{z}^{T}=\begin{array}{c} \underbrace{\frac{NI}{2\pi \left(x+R_{1}\right)}}+\\ incidend\;field\;H_{z0} \end{array}\;\begin{array}{c} \underbrace{\int_{0}^{a}\int_{0}^{b}\frac{4}{ab}f\left(w,v\right)\;\cdot \sum_{m=1}^{\infty }\sum_{n=1}^{\infty }\frac{\sin\left(\frac{m\pi x}{a}\right)\sin\left(\frac{\mathrm{n\pi y}}{b}\right)\sin\left(\frac{m\pi w}{a}\right)\sin\left(\frac{n\pi v}{b}\right)}{{\left(\frac{m\pi }{a}\right)}^{2}+{\left(\frac{n\pi }{b}\right)}^{2}-k^{2}}dwdv}\\ induced\;field\;H_{z} \end{array}\;\\ ,\;m,n=1,2,\cdots .\; \end{array}\; \end{array}$$

The distributions of the magnetic fields obtained here are similar to those obtained using the formulation given in [21,22]. In the next step, the electric field components are introduced using Maxwell’s equations, enabling the computation of electric and magnetic energy densities. The eddy field can be derived by substituting Equation (18) into the curl form of Maxwell’s equations (The electric field generated by the induced magnetic field has components in both the x- and y-directions of the cross-sectional area.):

(20) $$\begin{array}{c} E_{eddy}=\begin{array}{c} \underbrace{\frac{1}{\sigma +j\omega \varepsilon }.\frac{\partial H_{z}}{\partial y}}+\\ E_{x} \end{array}\;\begin{array}{c} \underbrace{\frac{-1}{\sigma +j\omega \varepsilon }.\frac{\partial H_{z}}{\partial x}}\\ E_{y} \end{array}.\; \end{array}$$

The corresponding electric and magnetic energies within the core are then given by the following:

(21) $$\begin{array}{c} E_{H}=\int_{V_{core}}\frac{1}{2}\mu^{\prime }{\left|H_{z}^{T}\right|}^{2}dv, \end{array}$$

(22) $$\begin{array}{c} E_{E}=\int_{V_{core}}\frac{1}{2}\varepsilon^{\prime }{\left|E_{eddy}\right|}^{2}dv. \end{array}$$

These expressions form the basis for evaluating the frequency-dependent electromagnetic energy distributions within the ferrite core.

## 3. Simulation and Analysis of Laminated Ferrite Cores

In addition to the analytical method discussed in Section 2, which is employed to solve the PDE of Equation (6) in electromagnetic fields, numerical methods such as the finite element method (FEM) and finite difference method (FDM) can also be utilized to model and solve the electromagnetic field distribution within the ferrite core. This section employs the three distinct computational approaches to calculate the magnetic field distribution under identical geometrical and excitation conditions, enabling both cross-validation of the results and evaluation of the applicability of each method.

**Figure 3:**
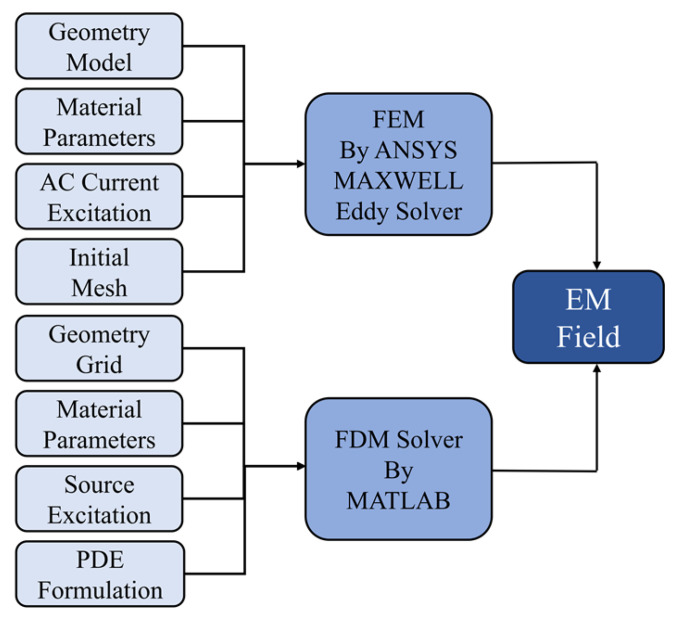
The basic solving workflow of FEM and FDM.

**Figure 4:**
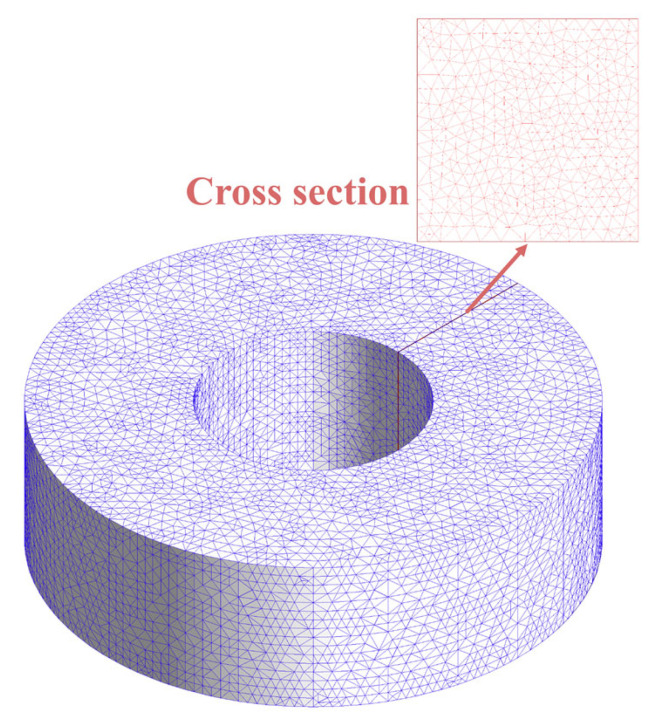
The result mesh of adaptive mesh iteration in Ansys Maxwell.

In Figure 3, the basic workflow obtained utilizing the FEM and FDM is presented. In the context of the FEM approach, the commercial software ANSYS Maxwell 2023R1 is utilized for the purpose of solving the eddy current problem. Although COMSOL is also widely used for similar simulations [23], ANSYS is adopted here due to its automatic adaptive meshing capability, which adjusts the mesh density based on energy error estimates until a user-specified accuracy is reached. The resulting mesh of the adaptive mesh iteration is illustrated in Figure 4. Conversely, the FDM method is implemented through the utilization of MATLAB R2022B scripts, within which the discretized PDEs are solved iteratively with finite orders. This approach has been demonstrated to provide good controllability and computational efficiency.

To better illustrate the specific calculation process of FDM and strictly capture the curvature effects of the toroidal geometry, the FDM simulation models the core in the cylindrical coordinate system (r,z), unlike the analytical derivation, which utilizes a Cartesian approximation. Consequently, the governing Equation (4) derived in Section 2 takes the form of the vector Helmholtz equation for the azimuthal magnetic field component $H_{\varphi }$:

(23) $$\begin{array}{c} \frac{\partial^{2}H_{\phi }}{\partial r^{2}}+\frac{1}{r}\frac{\partial H_{\phi }}{\partial r}-\frac{H_{\phi }}{r^{2}}+\frac{\partial^{2}H_{\phi }}{\partial z^{2}}+k^{2}H_{\phi }=-k^{2}H_{z0}, \end{array}$$

In Equation (4), the additional terms $\frac{1}{r}\frac{\partial H_{\phi }}{\partial r}$ and $\frac{H_{\phi }}{r^{2}}$ account for the radial variations inherent to the ring structure. The continuous domain is discretized into a grid with radial step ∆r and axial step ∆z. Utilizing the central difference scheme, the discretized equation at node (i,j) corresponding to coordinates ($r_{i},\;z_{j}$) is expressed as (five-point stencil)

(24) $$\begin{array}{c} \frac{H_{i+1,j}-2H_{i,j}+H_{i-1,j}}{{\left(\Delta r\right)}^{2}}+\frac{1}{r_{i}}\frac{H_{i+1,j}-H_{i-1,j}}{2\Delta r}-\frac{H_{i,j}}{r_{i}^{2}}+\frac{H_{i,j+1}-2H_{i,j}+H_{i,j-1}}{{\left(\Delta z\right)}^{2}}+k^{2}H_{i,j}=f_{i,j} \end{array}$$

Despite the similarities in the workflows of the FEM and FDM, there are notable differences in their modeling strategies and underlying solution mechanisms. The FEM has been demonstrated to be more appropriate for complex geometries and heterogeneous material distributions. Conversely, the FDM has been shown to excel in fast field computation within regular-shaped structures due to its explicit meshing and simplified formulation. Irrespective of the utilization of FEM or FDM, both methodologies facilitate the analysis of how differing input parameters affect the magnetic response of the core. This, in turn, improves the interpretability and credibility of the model.

To validate the proposed methods, a rigorous 3D Finite Element Method (FEM) simulation was conducted using Ansys Maxwell. Unlike the analytical and FDM approaches, the FEM model involves no geometric simplifications. The simulation setup ensures high fidelity through the following measures and details:

1. Boundary Conditions: The toroidal core is enclosed in a large cubic air domain (side length > 5 times the core radius). Zero-flux boundary conditions (Magnetic Insulation) are naturally applied to the outer surfaces of this domain. Due to the sufficient distance from the core, the electromagnetic field decays to negligible levels at these boundaries, effectively simulating an open-space environment.
2. Mesh Convergence: To accurately resolve the skin effect, the mesh is refined such that the maximum element size inside the ferrite is restricted to one-third of the skin depth (δ/3) at the maximum frequency.
3. Material Properties: The frequency-dependent complex permeability ($\mu^{\prime }-j\mu^{\prime \prime }$) experimentally extracted is imported into the Ansys material dataset, ensuring the material model strictly aligns with the physical samples.
4. Excitation: The excitation is provided by a single-turn current passing through the toroid center, driven by a harmonic current source satisfying Ampere’s Law.

**Figure 5:**
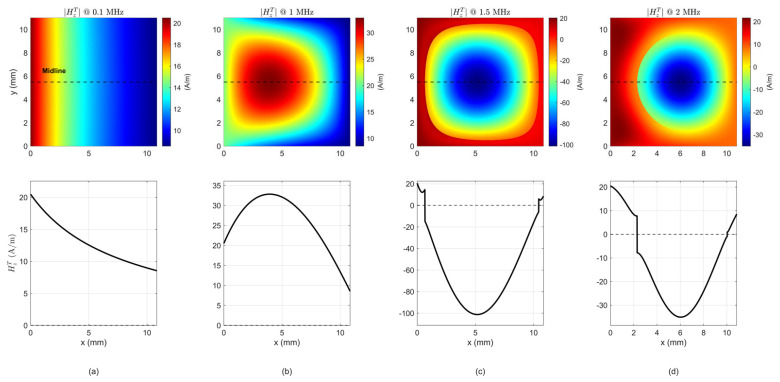
Distribution of the total magnetic field within the toroidal core cross-section at different frequencies. Top row: Total magnetic field $\left|H_{z}^{T}\right|$. Bottom row: Midline total magnetic field $H_{z}^{T}$ along the cross-section. (**a**) 0.1 MHz; (**b**) 1 MHz; (**c**) 1.5 MHz; (**d**) 2 MHz.

Utilizing the FDM approach, the magnetic field distribution across the rectangular cross-section of the ferrite core was solved at four distinct frequencies, incorporating both the incident and induced magnetic fields. As demonstrated in Figure 5, the initial row displays the distribution of the total magnetic field $\left|H_{z}^{T}\right|$ across the entire cross-section, while the subsequent row presents $H_{z}^{T}$ curves along the horizontal midline of the cross-section.

As demonstrated in Figure 5a, in the low-frequency range of 0.1 MHz and below, the induced magnetic field is found to be negligible. The flux distribution within the core is primarily governed by the incident field. Consequently, the total magnetic field is found to be almost entirely determined by Ampere’s Law, and no significant perturbation is observed at the core edges—characteristics that are typical of the quasi-static regime.

At a higher frequency of 1 MHz (see in Figure 5b), the induced field exhibits a marked tendency to concentrate within the central region of the core. This results in a constructive addition to the incident field along the z-axis, consequently leading to a significant increase in total magnetic flux. When the frequency is increased to 1.5 MHz and 2 MHz (see in Figure 5c,d), the induced field becomes dominant and local regions appear where it opposes the incident field, causing a reduction in total flux and revealing a cancellation trend. These observations suggest that the influence of the induced field on the overall distribution intensifies with frequency, thereby imparting a pronounced frequency dependence to the core’s inductive behavior.

It is noteworthy that the magnetic field at the core boundaries exhibited minimal variation across Figure 5, thereby substantiating the hypothesis that the edge region is pre-dominantly influenced by the induced field and consequently remains under the dominion of the incident field. This observation is in alignment with the Dirichlet boundary conditions delineated in Section 2.

Once the spatial magnetic field distribution within the core is known, the inductance can be obtained directly from its definition:

(25) $$\begin{array}{c} L=\frac{\Phi }{NI}=\frac{\int_{S}\mu H_{Z}^{T}ds}{NI}. \end{array}$$

The theoretical or ideal value of inductance, under the assumption that eddy currents are negligible (i.e., in low-frequency conditions as in Figure 5a), is given by

(26) $$\begin{array}{c} L_{ideal}=\frac{\mu N^{2}A_{e}}{l}. \end{array}$$

where $A_{e}$ represents cross-sectional area, and l represents average magnetic-path length. As previously discussed, the magnetic energy within the ferrite core is supplied by the EPC. When the magnetic energy equals the electric energy within the core, resonance occurs ($E_{E}(f_{r})=E_{H}(f_{r})$). The resonance frequency $f_{r}$ can be expressed as

(27) $$\begin{array}{c} f_{r}=\frac{1}{2\pi \sqrt{L\times EPC}}. \end{array}$$

Based on Equation (19), the magnetic field distribution within the ferrite core is determined by its physical dimensions and intrinsic material properties. Therefore, the following basic and essential parameters are specified for subsequent numerical simulations, as summarized in Table 1. The specific values for these parameters are selected to be consistent with those used in [7] to facilitate a direct comparison and validation of our results.

In addition, the product of the current and number of turns is set as *NI* = 1 (i.e., a single conductor carrying a 1 A current passes through the center of the toroidal core), in order to simulate the unsaturated condition of the core. It is also assumed that the ferrite material is isotropic and homogeneous.

**Figure 6:**
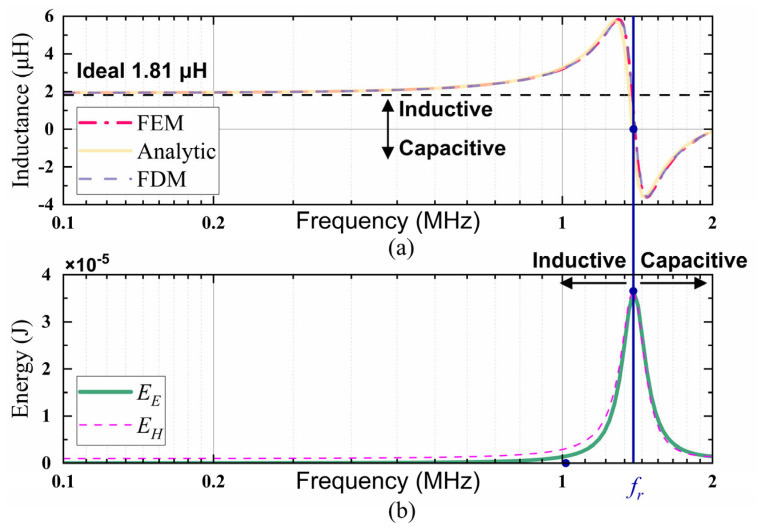
(**a**) Frequency-dependent inductance calculated results obtained from three methods: finite element method (FEM), finite difference method (FDM), and the analytical solution. (**b**) Evolution of magnetic and electric field energy as a function of frequency, calculated by using the analytical solution.

In Figure 6, the numerical calculation of the frequency-dependent inductance and electromagnetic energy within the ferrite core is presented, with the analytical method, FDM, and FEM simulations employed to derive these values. As shown in Figure 6a, the results from all three methods agree well over the tested frequency range, especially at a low frequency (e.g., <0.5 MHz), where the inductance values are consistent with the theoretical and ideal prediction from Equation (26), approximately 1.81 μH. This strong agreement confirms the accuracy of each method.

However, as the frequency increases, the manifestation of skin effects and dimensional resonance becomes evident. In order to further investigate this phenomenon, Figure 6b undertakes an analysis of the variation in electromagnetic energy distribution between magnetic and electric fields based on Equations (21) and (22). The results indicate that prior to the resonance frequency, magnetic energy dominates, indicating that the core exhibits inductive behavior. As the frequency increases, the strength of the electric field and the electric energy concomitantly increase, eventually reaching a state of equilibrium with the magnetic energy at resonance. At this frequency, the system transitions to a maximum energy-exchange state. Beyond this point, the electric energy exceeds the magnetic energy, and the entire ferrite core exhibits capacitive characteristics and a negative effective permeability. When interpreted from the perspective of energy, this trend unveils the underlying physical mechanism responsible for the frequency-dependent transition of ferrite materials from magnetic to capacitive behavior in the high-frequency regime.

To further examine, by simulation, how variations in core-cross-section geometry affect the inductive resonance characteristics, three toroidal configurations (#1–#3 in Table 2) were created. In each case, the average magnetic-path length ($l=\pi \left(R_{1}+R_{2}\right)$) was maintained at a constant value of 83 mm, and the effective cross-sectional area ($A_{e}=h\left(R_{2}-R_{1}\right)$) had a constant value of 120 mm^2^ to ensure an equal core volume was maintained. The thickness a and the height b were adjusted independently to obtain the three distinct cross-sectional shapes.

As can be seen in Table 2, the resonance frequency predicted by the three methods agrees closely for all three geometries and remains in good accordance with the reference data reported in [7]. This result reconfirms the consistency and accuracy of the three computational approaches when applied to different core shapes. Of particular interest is sample #3, which has a square core cross-section. In conditions of an identical magnetic-path length and effective area, the square cross-sectional sample yielded the lowest resonance frequency. In contrast, samples #1 and #2, which were flatter or more elongated, exhibited a higher resonance frequency. This observation is consistent with the theoretical findings in [7], which demonstrate that, for a given path length and area, a square cross-section maximizes electric-field energy storage inside the core and lowers the system’s resonance frequency.

**Figure 7:**
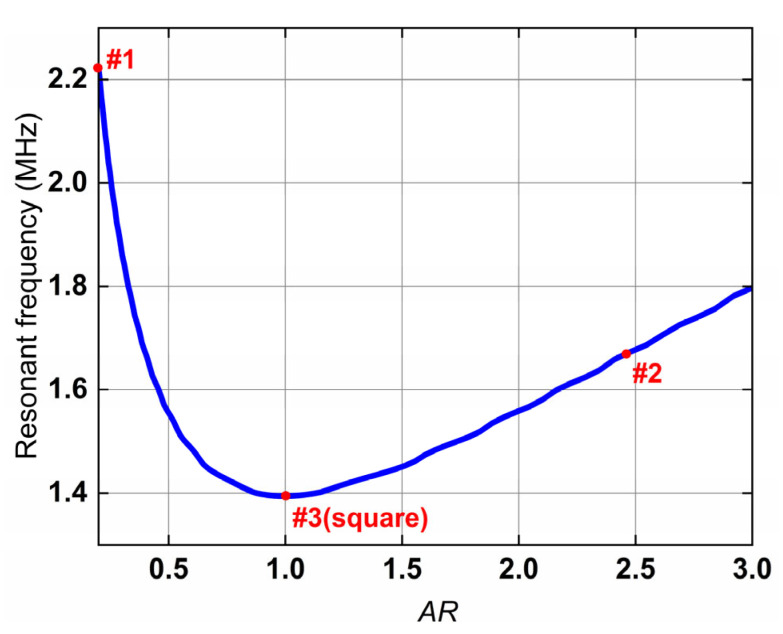
Resonant frequency as a function of the cross-sectional aspect ratio.

To further investigate and visually quantify the relationship between the cross-sectional geometry and the resonance frequency, we introduce a dimensionless aspect ratio (AR). This parameter is defined as the ratio of the cross-section’s width a (seen in Figure 1c) to its height b, i.e., AR=a/b. A parametric analysis was performed where the aspect ratio was varied while the total cross-sectional area $A_{e}$ was held constant to ensure a valid comparison. The resulting resonance frequency as a function of the AR is presented in Figure 7. As can be intuitively observed from Figure 7, the square cross-section (AR=1) yields the minimum resonant frequency. Therefore, in practical designs where a higher $f_{r}$ is desired, avoiding an aspect ratio close to unity is an effective approach to push the resonance higher.

An evaluation was conducted for the multi-layer laminated structure represented by samples #4 and #5. In this structure, the original core is divided axially into several identical segments while the overall magnetic-path length is kept unchanged. To model the multi-layer structures (samples #4 and #5), the analytical solution was applied to a single lamination. This was carried out by adjusting the height parameter in the model from the total height b to the single-layer height b/N. The governing Equation (19) is general for any rectangular cross-section and thus requires no modification. In such a configuration, it has been demonstrated that each segment retains the same dimensions, but the inter-layer insulation effectively forms multiple parallel eddy current loops. This, in turn, has been shown to reduce the overall electric-field energy, improve the inductive performance, and ultimately produce a marked increase in resonance frequency. For instance, the resonance frequency of sample #5 increases to 3.97 MHz, thereby demonstrating the efficacy of the laminated structure in augmenting high-frequency inductance.

The laminated structure reduces both the effective conductivity and the Equivalent Parallel Capacitance (EPC). Data in Table 2 clearly validates this dual benefit. According to the relationship $f_{r}\propto 1/\sqrt{L\cdot EPC}$ from Equation (27), this nearly threefold increase in $f_{r}$ strongly implies that the EPC was significantly reduced by the lamination.

This quantitative relationship between lamination count N, effective conductivity $\sigma_{eff}$, and Equivalent Parallel Capacitance (EPC) can be understood by examining two distinct physical mechanisms:

1. Impact on Effective Conductivity: As is well-established, laminating the core breaks the conductive paths for eddy currents. For a core of height *h* split into *N* laminations, the height of each conductive block becomes *h*/*N*. This suppresses large-scale eddy current loops, leading to a reduction in the bulk effective conductivity, which can be approximated as $\sigma_{eff}\propto 1/N$. This effect primarily reduces core losses at high frequencies.
2. Impact on Equivalent Parallel Capacitance (EPC): The dominant source of self-capacitance in bulk ferrite cores is their extremely high dielectric constant. When the core is laminated, thin insulating layers (such as oxidation) are introduced between the *N* ferrite blocks. These insulating gaps have a very low dielectric constant. This process effectively creates a new structure of *N* high-capacitance ferrite blocks connected in series with *N* − 1 very low-capacitance insulating gaps. In a series capacitor network, the total capacitance is dominated by the smallest capacitance. The total EPC can thus be expressed as the series combination shown in Equation (28):

(28) $$\begin{array}{c} \;\frac{1}{EPC_{total}}=\sum_{i=1}^{N}\frac{1}{EPC_{ferrite,i}}+\sum_{i=1}^{N-1}\frac{1}{C_{gap,i}}\approx \sum_{i=1}^{N-1}\frac{1}{C_{gap,i}}\; \end{array}$$

Assuming the *C_gap,i_* are similar, the total EPC is approximately *EPC_total_* = *C_gap_*/(*N* − 1). This establishes a second key relationship: the EPC is approximately inversely proportional to the lamination count *N* − 1.

Conversely, radial lamination of ferrite cores is seldom adopted for two primary reasons:

1. Achieving accurate alignment in practice is challenging, and maintaining manufacturing tolerances is difficult.
2. The necessity of creating a new mold for each distinct pairing of inner and outer radius results in a substantial increase in cost.

Although all three computational approaches (FDM, FEM, and the analytical method) yield resonance-frequency predictions that are in close agreement with the reference data, there is a substantial difference in their computational efficiencies. In Table 3, the time required for computation by each method is documented for a single frequency point.

As shown in Table 3, the FEM method is the most time-consuming approach. It is important to note that the FEM simulation presented here is performed on a full 3D model, serving as a rigorous benchmark to capture all potential geometric effects. In contrast, both the FDM and the analytical method solve the problem using 2D cross-sectional approximations. Therefore, the comparison in Table 3 does not represent direct algorithmic competition under identical conditions, but rather highlights the significant efficiency advantage gained by dimensionality reduction. The results confirm that for the toroidal core geometry, the proposed 2D methods can achieve an accuracy comparable to the 3D FEM benchmark while reducing the computation time by orders of magnitude. FDM attains nearly the same accuracy while achieving a noticeably higher speed, making it well-suited to regular geometries and homogeneous materials.

The analytical method under discussion is, in essence, still based on a truncated series expansion, with a finite-term discrete modal summation. It is notable that this method is capable of completing a single frequency point in just 9.2 s, which is considerably faster than the time taken for either numerical technique. These results confirm that, in circumstances where the geometry is regular and the electromagnetic boundaries are well defined, the analytical approach preserves accuracy while dramatically improving computational efficiency. This is highly advantageous for parameter sweeps and verification studies, especially in the case studied in this paper.

In the context of high-frequency magnetic-component modeling, where both accuracy and speed are paramount, the analytical solution offers the greatest computational advantage; FDM provides an efficient alternative for geometrically simple problems, whereas FEM remains the method of choice for complex materials, intricate structures, and multiphasic coupling scenarios.

## 4. Experimental Demonstration of High-Frequency Inductance Enhancement

In order to provide further validation of the simulation results presented in this paper regarding the enhancement of high-frequency inductance and magnetic energy storage capability via laminated ferrite core structures, a series of experimental samples were fabricated and tested. The outer and inner diameters of the samples are 27 mm and 11 mm, respectively, with three different core thicknesses of 8 mm, 4 mm, and 2 mm. The cores are then arranged in accordance with the desired configuration to assess their performance under varying levels of lamination. The Mn-Zn ferrite materials utilized for the samples were R5KZ, R7KC, and R10KZ (all supplied by DMEGC, China), with initial real parts of permeability of 5500, 7000, and 10,000, respectively.

**Figure 8:**
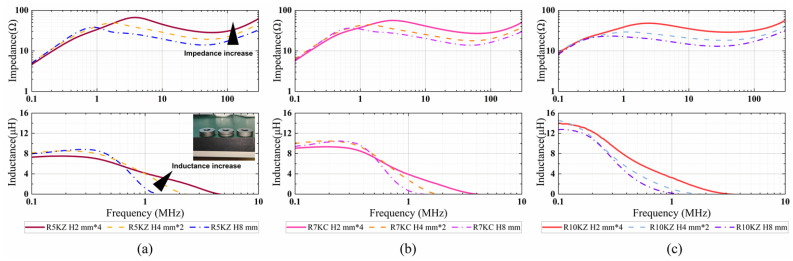
Measured impedance and inductance of ferrite cores with equal volume but different laminated configurations: monolithic core (8 mm height), two-layer laminated (4 mm × 2) cores, and four-layer laminated (2 mm × 4) cores with three kinds of Mn-Zn materials: (**a**) R5KZ; (**b**) R7KC; (**c**) R10K.

Impedance measurements were conducted using a HIOKI IM7581 Impedance Analyzer equipped with a HIOKI IM9202 Test Fixture (HIOKI E.E. Corporation, Ueda, Nagano, Japan). To ensure accuracy across the megahertz range, a standard Open-Short-Load (OSL) calibration was performed at the test port to establish a precise reference plane. Furthermore, to eliminate the parasitic effects of the test fixture and connecting wire harness, a compensation procedure was implemented. The residual series impedance and stray capacitance of the empty fixture were measured and mathematically de-embedded from the raw experimental data using the analyzer’s built-in functions, ensuring that the final results represent the intrinsic electromagnetic properties of the ferrite cores. Under room temperature conditions, the inductance and impedance of each sample were measured across a frequency range of 100 kHz to 300 MHz. The variation in impedance and inductance with frequency is shown in Figure 8.

As illustrated in Figure 8, under conditions of equal volume and material, laminated core structures exhibited a substantial enhancement in impedance and inductance performance in the high-frequency range when compared to monolithic counterparts. This effect became especially pronounced above 10 MHz, where the laminated structures demonstrated a higher magnetic performance. This result confirms that the laminated design effectively suppresses high-frequency magnetic losses, increases the resonant frequency, and thereby enhances the high-frequency inductance of the magnetic core. Lamination techniques remain a pivotal strategy for enhancing the high-frequency performance. As summarized in [24], dividing the magnetic core into laminated layers significantly mitigates eddy current losses and improves the quality factor in MHz-range inductive components.

When we further investigated the effect of different numbers of layers, it was found that the impedance and inductance values of the four-layer stacked samples were higher compared to the two-layer structure, indicating that the higher the number of layers, the better the performance. This observation confirms the trend found in the simulation results that the layer-stacked structure improves the high-frequency performance. Preliminary experimental validation confirms the hypothesis that the enhanced field uniformity along the stacking direction contributes to the improvement of high-frequency inductance and impedance. In addition, experiments were carried out to evaluate the impact of different ferrite materials with varying initial permeability on the magnetic performance. For example, low-frequency inductance increases with permeability (verified by Equation (26)), but the resonance frequency tends to decrease as inductance increases. Therefore, in practical designs, trade-offs between the stacking configuration and material permeability are necessary to achieve a balanced high-frequency performance.

**Figure 9:**
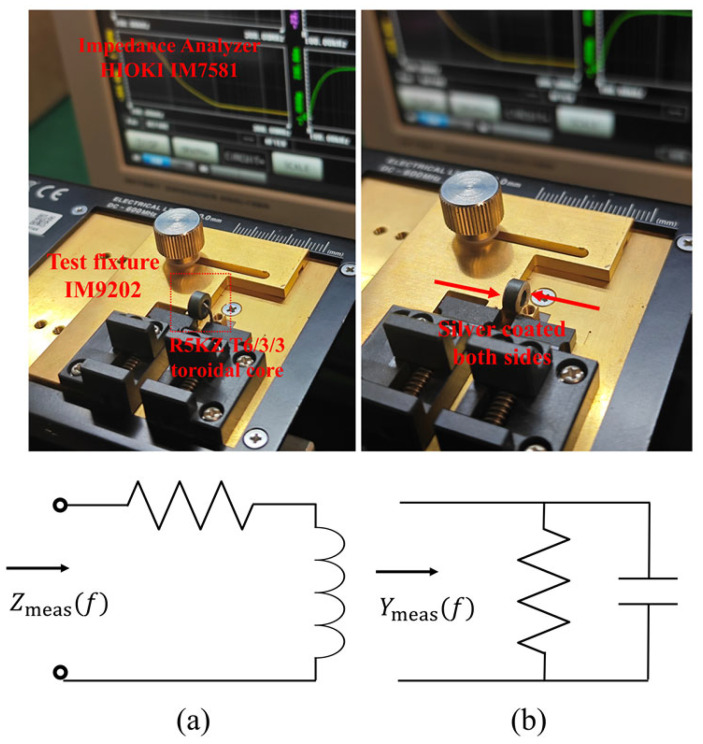
(**a**) Equivalent circuit and measurement setup for extracting complex permeability using the impedance method. (**b**) Equivalent circuit and measurement setup for extracting complex permittivity using the admittance method.

In order to validate the accuracy and practical relevance of the analytical method proposed in this work, an additional set of experiments was performed using R5KZ material. A small toroidal sample (with dimensions of 6 mm in outer diameter, 3 mm in inner diameter, and 3 mm in height) was selected for this study, as this configuration was found to minimize dimensional resonance and measurement distortion [12]. Following the calibration process, the complex impedance of the sample was measured using the impedance analyzer and the commercial test fixture, resulting in effective impedance (or admittance) curves. The impedance method was employed to extract the intrinsic complex permeability $\mu_{r}$ from $Z_{meas}(f)$, while the capacitance-admittance method was utilized to obtain the intrinsic complex permittivity $\varepsilon_{r}$. The equivalent circuit and measurement setup are illustrated in Figure 9. Subsequently, these parameters were entered into the analytical formulation to calculate inductance:

(29) $$\begin{array}{c} \mu_{r}=\mu_{r}^{\prime }-j\mu_{r}^{\prime \prime }=\frac{Z_{meas}l}{A_{e}j\omega }. \end{array}$$

It must be emphasized that silver electrodes must be coated on the top and bottom surfaces of the sample before measuring the admittance $Y_{meas}(f)$ for reliable conductivity extraction. Regardless of the flatness of the electrode plate or the pressure applied, the porous nature of the ferrite surface inevitably creates micro-air gaps at the contact interface. Consequently, these air gaps introduce significant measurement errors. To solve this problem, a solution was devised whereby a thin layer of highly conductive silver paste was applied to both electrode surfaces of the sample. This treatment has been shown to improve the uniformity of the electrode contact with the ferrite, thus improving the accuracy of the obtained data [25]. The intrinsic complex permittivity and conductivity were then calculated from the admittance data using the following expressions:

(30) $$\begin{array}{c} \varepsilon_{r}^{\prime }=\frac{Im\left(Y_{meas}\right)\cdot b}{\omega \varepsilon_{0}A_{e}}, \end{array}$$

(31) $$\begin{array}{c} \varepsilon_{r}^{\prime \prime }=\frac{Re\left(Y_{meas}\right)\cdot b}{\omega \varepsilon_{0}A_{e}}, \end{array}$$

(32) $$\begin{array}{c} \sigma =\frac{Re\left(Y_{meas}\right)\cdot b}{\omega \varepsilon_{0}A_{e}}\left[S/m\right]. \end{array}$$

**Figure 10:**
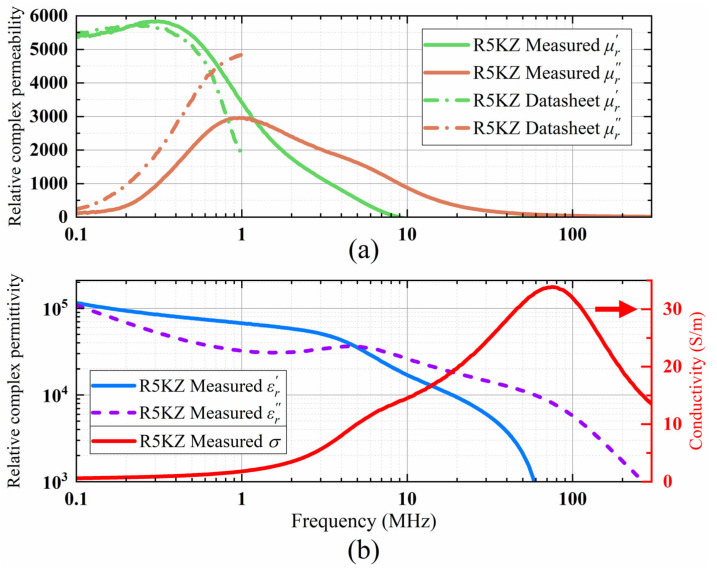
(**a**) Relative complex permeability $\mu_{r}$ versus frequency of R5KZ ferrite based on experiment and manufacturer’s datasheet. (**b**) Relative complex permittivity $\varepsilon_{r}$ and conductivity σ versus frequency of R5KZ ferrite based on experimental data.

The material characteristic parameters obtained using the above formulas are summarized in Figure 10. The disparities between the measured permeability and the manufacturer-provided data are primarily ascribed to variations in sample dimensions, since the reference values are generally obtained from standard samples with dimensions of 25 mm outer diameter, 15 mm inner diameter, and 8 mm height. This discrepancy in dimensions may consequently result in a systematic shift to the left of the measured permeability values [12]. Furthermore, it should be noted that, owing to constraints in the manufacturing process, there may be a discrepancy of up to ±25% between the initial permeability values of the final ferrite samples and the values specified in the datasheet.

**Figure 11:**
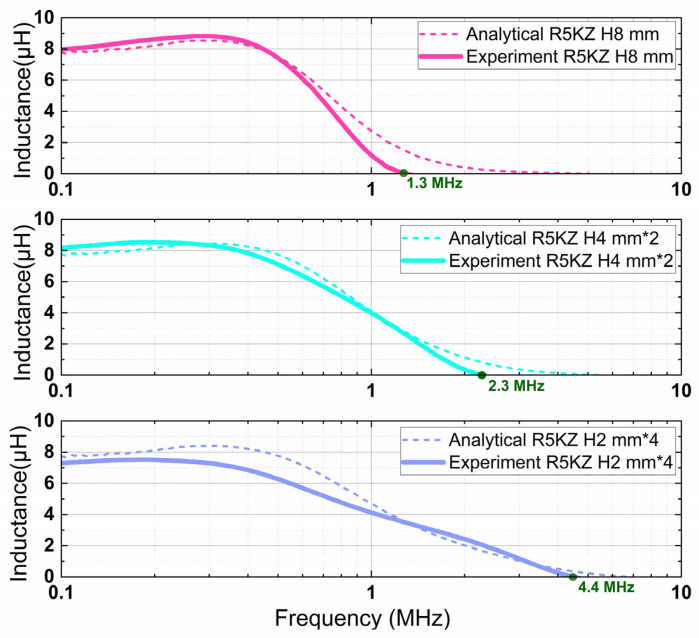
Comparison between analytical and experimental inductance results of R5KZ cores with identical volume but different laminated configurations: (Top) monolithic 8 mm core, (Middle) two-layer cores of 4 mm laminated, (Bottom) four-layer cores of 2 mm laminated. An upward shift in the resonance frequency is observed with increasing number of stacked layers.

Finally, the extracted complex permeability and permittivity were incorporated into the analytical model to compute the inductance of the magnetic core across a broad frequency spectrum. The calculated results were compared with experimental measurements, as shown in Figure 11. The analytical model closely tracks the measured impedance trends across the frequency range and accurately captures the key resonance frequency shift, thereby validating the effectiveness of the proposed simulation. Specifically, for R5KZ cores with an identical total volume but different laminated configurations, the resonance frequency shifts upward as the number of layers increases—from 1.3 MHz for the monolithic core to 2.3 MHz and 4.4 MHz for the two-layer and four-layer laminated cores, respectively. This clearly indicates that laminated structures are effective in reducing eddy current losses and significantly improving high-frequency inductive performance.

## 5. Conclusions

This study established a 2D analytical model for laminated Mn-Zn ferrite cores, demonstrating a significant computational efficiency advantage over time-consuming 3D FEM simulations. The model confirmed that a four-layer structure extends the bandwidth by increasing the resonant frequency by approximately 2.8 times. However, specific limitations must be acknowledged: the analytical solution employs a simplified Cartesian approximation that neglects curvature effects, and the Dirichlet boundary condition ($H_{ind}=0$) is strictly valid only for high-permeability materials. Furthermore, the experimental validation utilized physically stacked cores, which introduce uncontrolled parasitic capacitance distinct from industrial bonded laminations. Future work will address these constraints by incorporating curvature terms and quantifying industrial bonding effects. Additionally, investigations will extend to the influence of the laminated structure orientation, non-uniform thickness, and layer count to further advance the EMI application of laminated magnetic components.

## Figures and Tables

**Table 1:** Basic simulation parameters of the ferrite core.

Symbol	Description	Value	Unit
$R_{1}$	Inner radius	7.76	mm
$R_{2}$	Outer radius	18.61	mm
b	Height	11	mm
$\mu_{r}$	Relative permeability	1000	\
$\varepsilon_{r}$	Relative permittivity	2 × 10^5^	\
σ	Conductivity	2	S/m

**Table 2:** Comparison of resonant frequency calculated by different methods for various core dimensions.

Num.	Dimensions (mm) *R*_1_ × *R*_2_ × *b*	Geometry Sketch	*f_r_* (MHz) FEM	*f_r_* (MHz) Analytic	*f_r_* (MHz) FDM	*f_r_* (MHz) Reference [7]
#1	15.6 × 10.8 × 25	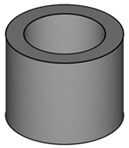	2.33	2.25	2.26	2.3
#2	21.7 × 4.6 × 7	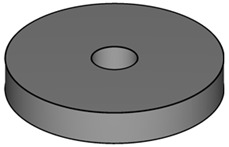	1.68	1.63	1.66	1.7
#3	18.6 × 7.7 × 11	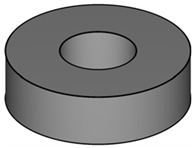	1.4	1.38	1.39	1.40
#4	2× 18.6 × 7.7 × 11/2	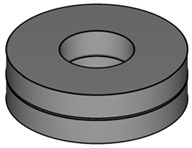	2.2	2.16	2.17	2.16
#5	4× 18.6 × 7.7 × 11/4	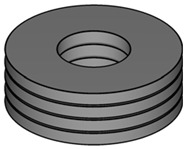	3.98	3.98	3.99	3.97

**Table 3:** Computation time comparison of three methods.

Method	Mesh	Computation * Time (s/Single Frequency)
FDM	1000 × 1000 (2D)	29.1
FEM	5.9 × 10^5^ (3D, Tetrahedral Mesh)	300
Analytical Method	2000 × 2000 (2D, m = n = 50)	9.2

* Hardware and software specifications: Intel Xeon(R) Gold 6242R CPU@3.10 GHz, 32 GB memory, ANSYS ADET 2023R1 with HPC and MATLAB R2022B.

## Data Availability

Data are contained within the article.
